# Two new mixed-ligand coordination polymers based on multi-N chelating ligand inhibit YAP expression and induce caspase-mediated spinal tumor cell apoptosis

**DOI:** 10.1590/1414-431X20198499

**Published:** 2019-05-16

**Authors:** Yi Fu Sun, Li Wei Shao, Qi Chen, Xu Gao, Fang Li, Chang Yan Wu

**Affiliations:** Department of Orthopedics, China-Japan Union Hospital of Jilin University, Changchun, Jilin, China

**Keywords:** Coordination polymers, Glioma cell strains, Cytotoxicity, Apoptosis, Immunoblotting

## Abstract

Two new coordination polymers [Zn (bdc)(bpybzimH_2_)](DMF)_0.5_ (1, H_2_bdc=1,4-dicarboxybenzene, bpybzimH_2_=6,6′-bis-(1H-benzoimidazol-2-yl)-2,2′-bipyridine, DMF=N,N-dimethylformamide) and [Co (bpybzimH_2_)(sbc)]H_2_O (2, H_2_sbc=4-mercaptobenzoic acid) have been successfully prepared under solvothermal conditions using the multi-N chelating organic ligand bpybzimH_2_ as the foundational building block. In addition, the Cell Counting Kit-8 assay was conducted to evaluate the anti-proliferation activity of compounds 1 and 2 against human spinal tumor cells OPM-2. The cell viability curves showed that the two compounds have anti-proliferation activity on spinal tumor cells, and the activity of compound 1 is higher than compound 2. The annexin V-FITC/PI assay and western blot were used to detect the apoptotic percentage of OPM-2 cells incubated with compounds 1 and 2. The YAP protein expression and its role in cell apoptosis were further studied with qRT-PCR, immunoblotting, and flow cytometer.

## Introduction

New drugs for the treatment of cancer with better rates of cure and side effects that are less severe are greatly needed since cancer has become one of the most serious diseases that kills millions of people every year (1). In this field, coordination chemistry has a great potential to offer a wide variety of compounds with different geometry, redox reactivity, and a diversity of mechanisms related to DNA binding, some of them unique to metals (2). The usefulness of coordination metal complexes in cancer chemotherapy has been demonstrated by cisplatin and other platinum coordination compounds, which are amongst the most successfully used anticancer drugs, but this is severely limited by the serious side effects, general toxicity, and drug resistance (3–5). The apparent clinical problems of recent chemotherapeutic drugs have led to the development of novel anticancer agents based on the use of not only essential metallic elements, but also of coordination polymers with diversiform structures (6).

Since the discovery of coordination polymers (CPs), these materials have been intensively studied due to their potential applications in areas such as luminescence, gas adsorption, and cytotoxic agents (7–10). Within these systems, we could consider metal-organic chains (MOCs) as 1D-coordination polymers in which the combination of metal centers and organic ligands provides fantastic possibilities for the construction of materials with various structures and functionalities. Furthermore, the concept of mixed-ligand synthesis has been applied to generate multiple-component coordination polymers that exhibit distinct properties with respect to the pure form (11). According to the literature, a large variety of coordination compounds based on multi-N donor ligands have been synthesized with interesting biological properties, which could be applied as anticancer reagents against various cancer cells (12–15). For these reasons, we decided to synthesize new MOCs by using zinc and cobalt as metal ions in order to study some of the anti-cancer properties or activity exhibited by these materials. On another level, we selected 6,6′-bis-(1H-benzoimidazol-2-yl)-2,2′-bipyridine (bpybzimH_2_) as ligand, a scarcely explored molecule for CPs construction, given its potential capacity to fulfill the above-mentioned structural and biological roles (16). Regarding its coordinating ability in metal-organic architectures, it is necessary to introduce another organic ligand to extend its dimension.

**Figure 1:**
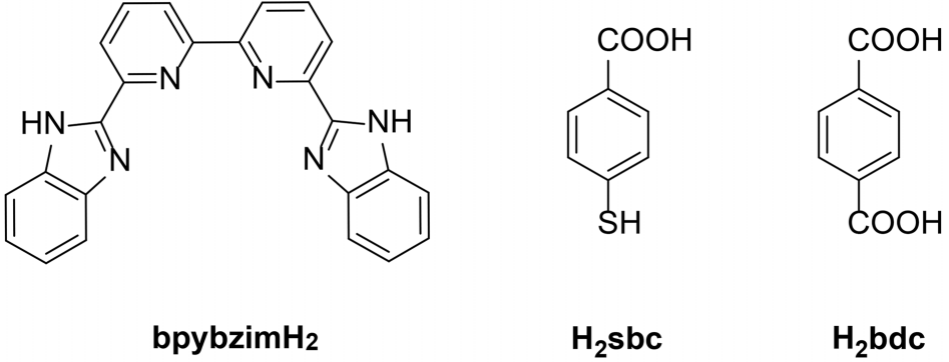
Chemical drawings for the organic ligands in this study.

In this study, by using the mixed-ligand approach, two new coordination polymers [Zn (bdc)(bpybzimH_2_)](DMF)_0.5_ (1, H_2_bdc=1,4-dicarboxybenzene, bpybzimH_2_=6,6′-bis-(1H-benzoimidazol-2-yl)-2,2′-bipyridine, DMF=N,N-dimethylformamide, Figure 1) and [Co (bpybzimH_2_)(sbc)]H_2_O (2, H_2_sbc=4-mercaptobenzoic acid) have been successfully prepared under solvothermal conditions using the multi-N chelating organic ligand bpybzimH_2_ as the foundational building block. Then, we evaluated the anti-proliferation activity of compounds 1 and 2 against human spinal tumor cells OPM-2.

## Material and Methods

### Chemicals and instruments

All reagents and solvents were used as received from commercial suppliers without further purification. The organic ligands used in this work were obtained from Jinan Henghua Sci. & Tec. Co. Ltd. (China) without further purification. FT-IR spectra were recorded within the 4000–400 cm^–1^ wave-number range using a Bruker TENSOR 27 Fourier transform infrared spectrometer (Germany) with the KBr pellet technique and operating in the transmittance mode. The elemental analyses were performed with PerkinElmer 240 CHN analyzers from Galbraith Laboratories, USA.

The human spinal tumor cells OPM-2 and normal human embryonic kidney cell line HEK-293 were purchased from American Type Culture Collection (ATCC, USA). The OPM-2 cell line was cultured in RPMI-1640 medium (Gibco, Life Technologies, USA); the HEK-293 cell line was cultured in Dulbecco's modified Eagle's medium (DMEM; Gibco, Life Technologies). Both culture mediums were supplemented with 100 U/mL penicillin and 100 U/mL streptomycin solutions (Gibco, Life Technologies), 10% (v/v) heat-inactivated fetal bovine serum (FBS) (HyClone, USA), and 2% L-glutamine. All the cells were cultured at 37°C in a humidified atmosphere of 5% CO_2_ with 95% air. The culture medium was replaced twice a week according to the cell growth status.

### Preparation of [Zn (bdc)(bpybzimH_2_)](DMF)_0.5_ (1) and [Co (bpybzimH_2_)(sbc)]H_2_O (2)

A mixture of Zn (NO_3_)_2_·6H_2_O (30 mg, 0.1 mmol), 6,6′-bis-(1H-benzoimidazol-2-yl)-2,2′-bipyridine (bpybzimH_2_, 47 mg, 0.12 mmol), H_2_bdc (0.1 mmol, 16 mg), DMF (4 mL), and H_2_O (2 mL) was added to a 20-mL glass vial. The vial was placed in an oven and kept at 100°C for three days. After cooling to room temperature, colorless crystals of 1 were obtained by filtration, washed with EtOH, and dried in air. Analog calculation for C_33.5_H_23.5_N_6.5_O_4.5_Zn: C, 61.48; H, 3.62; N, 13.91; Found: C 61.36, H 3.52, N 13.86 %. IR (KBr pellet, cm^−1^): 3445m, 3126w, 3011s, 2968m, 2876w, 1701m, 1614s, 1523s, 1422s, 1342m, 793m, 642m.

A mixture of Co (NO_3_)_2_·6H_2_O (30 mg, 0.1 mmol), 6,6′-bis-(1H-benzoimidazol-2-yl)-2,2′-bipyridine (bpybzimH_2_, 47 mg, 0.12 mmol), 4-sulfanylbenzoic acid (H_2_sbc, 0.1 mmol, 15 mg), DMF (4 mL), EtOH (2 mL), and H_2_O (0.5 mL) was added to a 20-mL glass vial. The vial was placed in an oven and kept at 80°C for three days. After cooling to room temperature, deep purple crystals of 2 were obtained by filtration, washed with EtOH, and dried in air. Analogue Calculation for C_31_H_22_CoN_6_O_3_S: C: 60.29; H: 3.59; N: 13.61. Found: C 59.99, H 3.62, N 13.79%. IR (KBr pellet, cm^−1^): 3121m, 3042w, 2943s, 2867w, 1698s, 1559w, 1502s, 1364s, 1277s, 1123s, 1026s, 804m, 692s.

### Crystal structure determination

The X-ray single crystal diffraction data of 1 and 2 were collected on a Bruker D8 diffractometer with an Apex II detector (Brucker). Data collection and reduction were performed using Apex II software suite (17). The structure was solved using direct methods followed by least-squares on *F* ^2^ using SHELXTL (18). All hydrogen atoms attached to oxygen and carbon were generated geometrically and all non-hydrogen atoms were refined anisotropically. Crystals of 2 were found to be a partial merohedral twins, and the intensity of reflections was separated into two components to produce the HKLF 4 and HKLF 5 files using the CrysAlis PRO software (Agilent, 2011, USA). The initial structure was solved by direct methods from the HKLF4 data and refined on an HKLF 5 file, and therefore, the merging of equivalent reflections was switched off using MERG 0. The number of unique reflections and the *R*_int_ are taken from the refinement of the corresponding HKLF 4 file and are thus approximations. Crystallographic data are summarized in Table 1.

**Table 1:** Crystal data and structure refinements for compounds 1 and 2.

Identification code	1	2
Empirical formula	C_67_H_47_N_13_O_9_Zn_2_	C_31_H_22_CoN_6_O_3_S
Formula weight	654.46	617.54
Temperature/K	288.0(3)	293(2)
Crystal system	triclinic	monoclinic
Space group	P-1	P2_1_/n
a/Å	11.4304 (9)	14.0294 (9)
b/Å	12.1861 (9)	9.8250 (6)
c/Å	12.7626 (11)	19.9761 (12)
α/°	111.901 (8)	90.00
β/°	96.713 (7)	105.962 (7)
γ/°	98.612 (7)	90.00
Volume/Å^3^	1601.7 (2)	2647.3 (3)
Z	2	4
ρ_calc_g/cm^3^	1.357	1.549
μ/mm^–1^	0.816	0.775
Reflections collected	21544	4902
Independent reflections	5958 [R_int_ = 0.0726]	4902 [R_int_ = 0.0000]
Data/restraints/parameters	5958/0/388	4902/2/380
Goodness-of-fit on F^2^	1.112	1.217
Final R indexes [I>=2σ (I)]	R_1_ = 0.0744, wR_2_ = 0.2135	R_1_ = 0.0773, wR_2_ = 0.2215
Final R indexes [all data]	R_1_ = 0.1123, wR_2_ = 0.2307	R_1_ = 0.0904, wR_2_ = 0.2280
Largest diff. peak/hole/e Å^-3^	1.28/-0.37	0.63/-0.64
CCDC	1891771	1891772

### Viability of cells

To assess the viability of OPM-2 spinal tumor cells and human embryonic kidney HEK-293 cells (normal human cells) after treatment with compounds 1 and 2, the Cell Counting Kit-8 (CCK-8) assay was performed according to the manufacturer's protocols (19). Briefly, the OPM-2 cells were seeded into 96-well plates at a density of 5×10^5^ cells/well with a total volume of 100 µL medium per well. All the cells were cultured at 37°C in a humidified atmosphere of 5% CO_2_ with 95% air for 24 h. Then, the cells were incubated with a series of concentrations (1, 2, 4, 8, 10, 20, 40, 80, 100 μM) of compounds 1 and 2, for 24 h. After incubation, the culture medium was discarded and the 10% CCK-8 (Dojindo Laboratories, Japan) in 100 μL RPMI-1640 medium without FBS was added into wells for 2 h incubation at 37°C in the dark. Finally, the absorbance of each well was measured with a microplate reader (ELX808; BioTek, USA) at 450 nm. The cell viability curves were calculated and plotted. The half-maximal inhibitory concentration (IC_50_) values were calculated using SPSS version 22.0 (IBM, USA). In all of these experiments, three replicate wells were used to determine each point.

### Annexin V-FITC/PI apoptosis analysis

For the apoptosis analysis, the OPM-2 cells were stained with annexin V-FITC/PI (BD Biosciences, USA) following the manufacturer's instruction (20). In brief, the OPM-2 cells were seeded in 6-well plates (1×10^6^ cells/well) at 37°C, 5% CO_2_ overnight. After the cells reached logarithmic growth with the confluence reaching 70–80%, the OPM-2 cells were treated with compounds 1 and 2 for 24 h. The same volume of solvent (negative control) and oxaliplatin (positive control) was added into the wells for incubation. Twenty-four hours later, the cells were trypsinized, washed 3 times with pre-cooled PBS, and re-suspended in 500 μL annexin V binding buffer. Then, the OPM-2 cells were incubated with 5 μL annexin V-FITC and 5 μL propidium iodide (PI) solution for 15 min at 37°C in the dark. The OPM-2 cells apoptosis was analyzed by flow cytometry (BD Via, USA) at an excitation wavelength of 488 nm and emission wavelengths of 525 and 625 nm. The results were analyzed using flow cytometry (FACSCalibur, BD Biosciences, USA). Each experiment was performed in triplicate.

### Transfection

The small interfering RNA (siRNA) oligonucleotide sequence targeting *yap* gene, a key effector in hippo pathway, was designed and synthesized by Shanghai GenePharma (China). The *yap* overexpression plasmid was stored in our laboratory. Twenty-four hours before transient transfection, the OPM-2 cancer cells were collected and seeded into 6-well plates at a density of 5×10^5^ cells/well. When cell growth was in the logarithmic stage with the confluence reaching 70–80%, the *yap*-siRNA and plasmid were transfected into the OPM-2 cells using Lipofectamine TM3000 Transfection Reagent (Invitrogen, USA) according to the manufacturer's protocol (21). The negative control group was transfected with negative control siRNA and vector plasmid by the same method. Efficiency of gene silencing was detected 48 h after the transfection with qRT-PCR and western blot.

### qRT-PCR assay

The mRNA expressions of hippo pathway in OPM-2 cancer cells after treatment with compounds 1 and 2 were measured using quantitative reverse transcription polymerase chain reaction (qRT-PCR) according to the protocol (22). Total RNA in OPM-2 cells was extracted using TRIzol™ Plus RNA Purification Kit (Invitrogen) according to the manufacturer’s instructions, and then the quality of RNA was evaluated using the OD260/OD280 ratio. The cDNA was synthesized using High-Capacity cDNA Reverse Transcription Kit (Applied Biosystems, USA). The PCR primer sequences in this experiment are listed in Table 2. The PCRs were conducted using the qRT-PCR miRNA Detection Kit (Invitrogen): 95°C for 15 min, followed by 40 cycles of denaturation at 95°C for 5 s, annealing at 55°C for 30 s, and extension at 72°C for 30 s. Each experiment was performed in triplicate and the relative quantification was analyzed by the 2^–ΔΔCt^ method.

**Table 2:** Primers and si-RNA sequences.

Name	Sequence
*yap*	CCCTCGTTTTGCCATGAACC GTTGCTGCTGGTTGGAGTTG
*gapdh*	AATGGGCAGCCGTTAGGAAA GCGCCCAATACGACCAAATC
*yap*-siRNA	GGCAGACUGAAUUCUAAAUUUUUCCGUCUGACUUAAGAUUUA
Control-siRNA	UUCUCCGAACGUGUCACGUTTACGUGACACGUUCGGAGAATT

### Western blot

After treatment of OPM-2 cells with different concentrations of the tested compounds for 24 h incubation, the cells were harvested and lysed with the cell lysis buffer (1% NP-40, 1% sodium dodecyl sulfate (SDS), 150 mM NaCl, 25 mM Tris-HCl (pH 7.6), 1% deoxycholic acid sodium salt, 1% PMSF) for 30 min on ice. Total proteins in OPM-2 cells were isolated using M-PER™ Mammalian Protein Extraction Reagent (Thermo Fisher Scientific, USA). The concentration of total proteins was quantified using BCA Protein Assay Kit (Beyotime Biotechnology, China) according to the protocol. Then, an equal amount of protein was denatured and separated by sodium dodecyl sulfate polycrylamide gel electrophoresis (SDS-PAGE), and transferred to a 0.22 mm nitrocellulose membrane. The membrane was blocked with PBS containing 5% nonfat milk for 2 h at room temperature and incubated with primary antibody at 4°C overnight, followed by secondary antibody for 2 h. The immunoblots were visualized by enhanced chemiluminescence kit (Thermo Fisher Scientific).

### Statistical analysis

All data are reported as means±SD from three independent experiments. The statistical analyses were carried out for measurement data in GraphPad Prism 5.0 (USA). The differences were considered significant at P<0.05.

## Results and Discussion

### Molecular structure of 1 and 2

**Figure 2:**
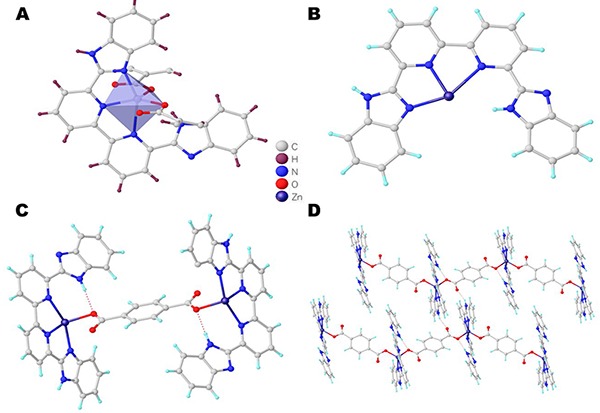
**A**, Molecular unit of complex 1. **B**, Binding pattern for the bpybzimH_2_ ligand. **C**, Binding pattern for the bdc^2-^ ligand. **D**, H-binding interaction between the adjacent chains.

The pristine coordination polymer 1 was obtained through the solvothermal reaction of Zn (NO_3_)_2_·6H_2_O, H_2_bdc, and bpybzimH_2_ in a mixed solvent of DMF and water at 120°C for three days. Single crystal X-ray diffraction analysis revealed that compound 1 crystallized in the triclinic crystal system, space group P-1, and had a 1D chain-like network. The asymmetric unit of 1 was composed of one crystallographic independent Zn (II) ion with full occupancy, two half bdc^2-^ ligands, one bpybzimH_2_ ligand, and a half DMF molecule, all of which contributed to a neutral framework structure. As shown in Figure 2A, the central Zn (II) atom is five-coordinated with two O atoms from two different bdc^2-^ ligands and three N atoms from one bpybzimH_2_ ligand, shaping a [ZnO2N3] disordered trigonal bipyramidal coordination surrounding. The Zn (II)-O bond distances were in the range of 1.991(2) to 1.944(2) Å and the Zn (II)-N bond lengths were in the range of 2.045(3) to 2.382(3) Å, all of which were in the normal range of Zn (II)-based coordination polymers constructed from the H_2_bdc and N-donor pyridyl ligands (23). The bpybzimH_2_ ligand acted as a chelating ligand that locked the single Zn (II) ion using its two pyridyl N atoms and one imidazolyl N atom along the ac plane, and the formed [ZnbpybzimH_2_]^2+^ molecular building block was further connected with each other via the bdc^2-^ ligand (Figure 2B). It is worth noting that only three N atoms in the bpybzimH_2_ took part in the coordination, and the residue uncoordinated N atom had an H atom on its axis site with an H-bonding interaction observed between the N-H atoms and the carboxylic O atom on the bdc^2-^ ligand. As for the bdc^2-^ ligand, it showed a linear configuration with paralleling carboxylate groups, and both carboxylate groups adopted monodentate coordination mode to link two Zn (II) ions separated by a distance of 11.124 Å (Figure 2C). The 1D chain-like structures were further extended into a 3D supramolecular network via the H-bond interactions (N-H-O, Figure 2D). The structure of 1 was similar to the reported {Zn (bdc)(phen)(H_2_O)}_n_ based on the same bdc^2-^ linker and the N-chelating 1,10-phenanthroline ligand, indicating the combination of linear carboxyl linkers and N-donor chelating ligands tended to afford the 1D chain-like networks (23).

**Figure 3:**
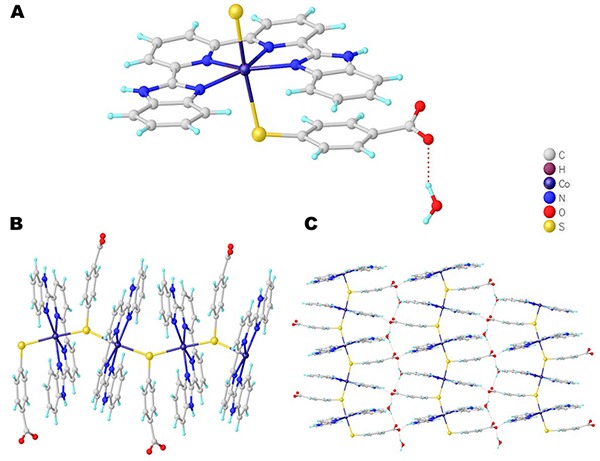
**A**, Molecular unit of complex 2. **B**, 1D chain-like network for complex 2. **C**, 3D supramolecular unit of complex 2 via H-binding interaction.

Single-crystal X-ray diffraction analysis revealed that complex 2 belonged to the monoclinic crystal system, space group P2_1_/n, and revealed a 1D chain-like network. The fundamental unit of 2 contained one central Co (II) ion, one bpybzimH_2_ ligand, one sbc^2-^ ligand, and one lattice water molecule, all of which contributed to a neutral network structure. As shown in the Figure 3A, the Co (II) atom performed a six-coordinated geometry by four N atoms from one bpybzimH_2_ ligand and two S atoms from two different sbc^2-^ ligands, forming a distorted octahedral geometry. The Co (II)-N bond distances were in the range of 2.123(1) to 2.129(2) Å and the Co (II)-S bond distances were in the range of 2.504(2) to 2.645(2) Å. The bpybzimH_2_ ligand chelated with the Co (II) ion using its two pyridyl N atoms and two imidazolyl N atoms to afford the [Co-bpybzimH_2_]_2_ molecule unit, which is further connected with the µ_2_-bridging S atom on the sbc^2-^ ligand to give rise to a l D chain-like network along the b axis (Figure 3B). As for the sbc^2-^ ligand, it only used its S atoms to act as a two-connected node, leaving the deprotonated carboxylic group uncoordinated, which further formed H-binding interaction with the lattice water molecule. The adjacent 1D chains were further extended into the 2D supramolecular architecture via the H-bonding interaction (Figure 3C). According to the literature, the cobalt(II)-based coordination compounds based on the H_2_sbc ligand have not been reported so far (24).

**Figure 4:**
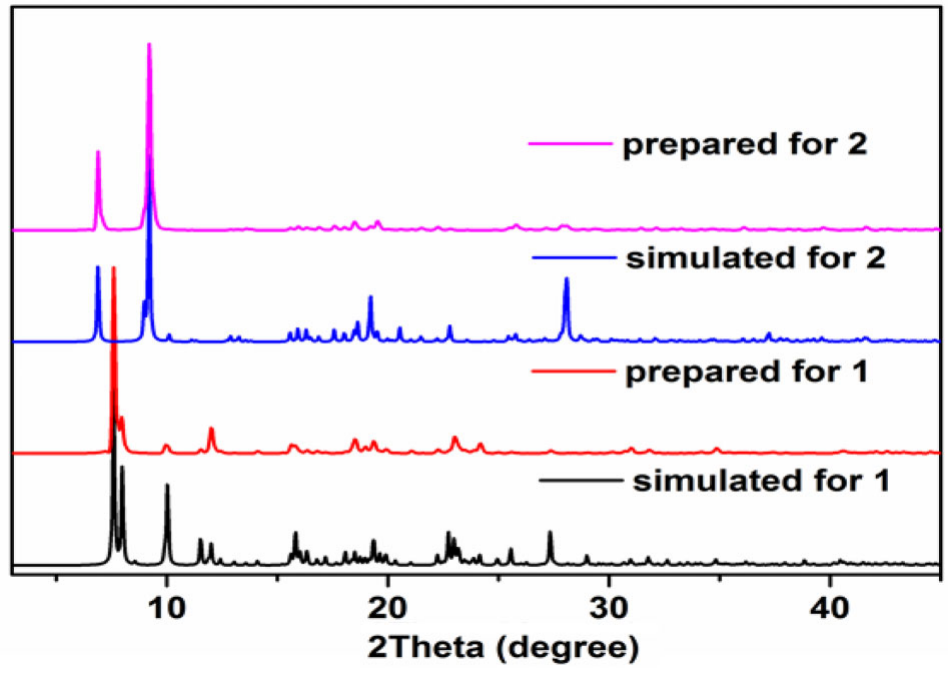
PXRD patterns for complexes 1 and 2.

The phase purity of the as-prepared complexes 1 and 2 has been confirmed via the PXRD measurements at room temperature using their bulky crystalline samples. As shown in Figure 4, the well-defined diffraction peaks revealed the high crystallinity of the products, which are in agreement with the simulated patterns from the crystal data.

### Anti-proliferation activity of compounds on cancer cells

**Figure 5:**
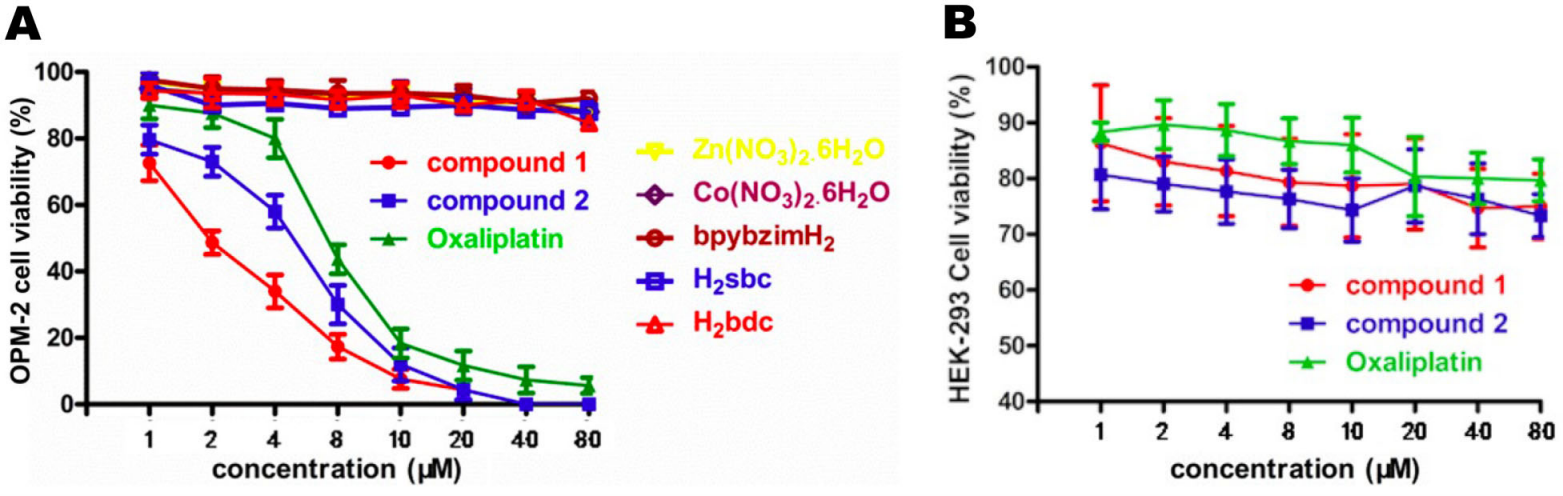
Compounds 1 and 2 inhibited OPM-2 cell proliferation. **A**, Cell viability curves were measured by CCK8 assay after treatment with compounds 1 and 2, Zn (NO_3_)_2_·6H_2_O, Co (NO_3_)_2_·6H_2_O, bpybzimH_2_, H_2_sbc, and H_2_bdc for 24 h. **B**, The half inhibitory concentration (IC_50_) of compounds 1 and 2 against HEK-293 cells. Data are reported as means±SD.

The *in vitro* antitumor capabilities of compounds 1 and 2 as well as their starting materials (metal ions and organic ligands) were evaluated with the CCK8 assays. The absorbance values at 570 nm reflected the cancer cells viability after treatment. As shown in Figure 5, the OPM-2 cell viability decreased in a concentration-dependent manner when treated with compounds 1 and 2. The inhibitory activity of compound 1 was obviously stronger than compound 2. It should be noted that the organic ligands (H_2_sbc, H_2_bdc, and bpybzimH_2_) and the metal ions [Zn (NO_3_)_2_·6H_2_O and Co (NO_3_)_2_·6H_2_O] showed negligible effect on the cell viability of OPM-2 cells, indicating the organic ligands used in this study showed no anticancer activity. This comparative study also showed that the chelating of the organic ligands with the metal ions might account for the observed anticancer activity.

The IC_50_ values are listed in Table 3. The IC_50_ of compound 1 in OPM-2 cells was 1.9±0.05 µM, which was significantly lower than the IC_50_ (5.2±0.1 µM) of compound 2. Even though, these two compounds had lower IC_50_ compared to the positive control drug oxaliplatin. This indicated that compounds 1 and 2 showed excellent anti-cancer effects.

**Table 3:** Half inhibitory concentration values of compounds 1 and 2, and oxaliplatin against OPM-2 cancer cells and normal human HEK-293 cells

Cell/drug	Compound 1 (%)	Compound 2 (%)	Oxaliplatin (%)
OPM-2	1.9 ± 0.05	5.2 ± 0.1	7.8 ± 0.2
HEK-293	>80	>80	>80

Data are reported as means±SD of three independent experiments.

### Compounds induced apoptotic cell death

**Figure 6:**
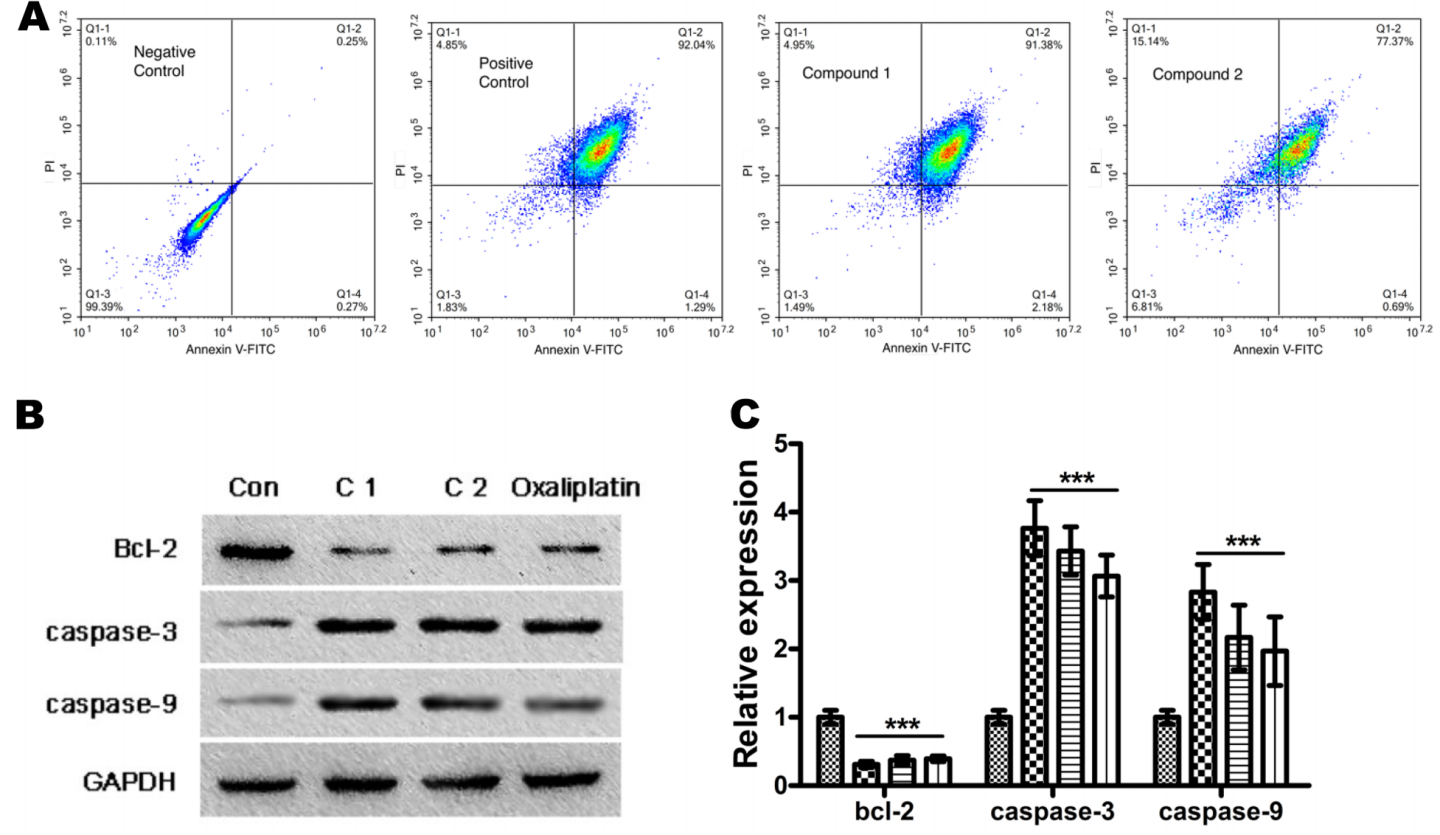
Effects of compounds 1 and 2 on OPM-2 cell apoptosis. **A**, Apoptotic OPM-2 cells were detected by flow cytometry following annexin V-FITC/PI double staining. **B**, The protein expression of Bcl-2, cleaved caspase-3, and cleaved-caspase-9 was detected by western blot. **C**, Statistical results of apoptotic protein expression. Representative results of three experiments are shown. Data are reported as means±SD. ***P<0.001 compared to control (small squares column) (ANOVA).

Most anti-cancer drugs exert their effects by elevating the production of apoptosis in cells, so we assessed whether the anti-proliferation activity of compounds 1 and 2 is also mediated by cancer cell apoptosis by Annexin V-FITC/ PI double staining assay and quantitatively evaluated the apoptotic percentage in OPM-2 cells via flow cytometer. As shown in Figure 6A, after incubation with compounds 1 and 2, the rate of apoptotic cells was significantly increased (91.38±3.9 and 77.37±2.8%, respectively). Moreover, the activity of compound 1 was significantly better than that of compound 2.

Furthermore, the western blot analysis showed that the key markers of cell apoptosis cleaved caspase-3 and cleaved caspase-9 were elevated compared with the control group (Figure 6B). All the results indicated that the compounds caused the production of apoptotic OPM-2 cell death.

### Compounds induced cell apoptosis via downregulating YAP expression

**Figure 7:**
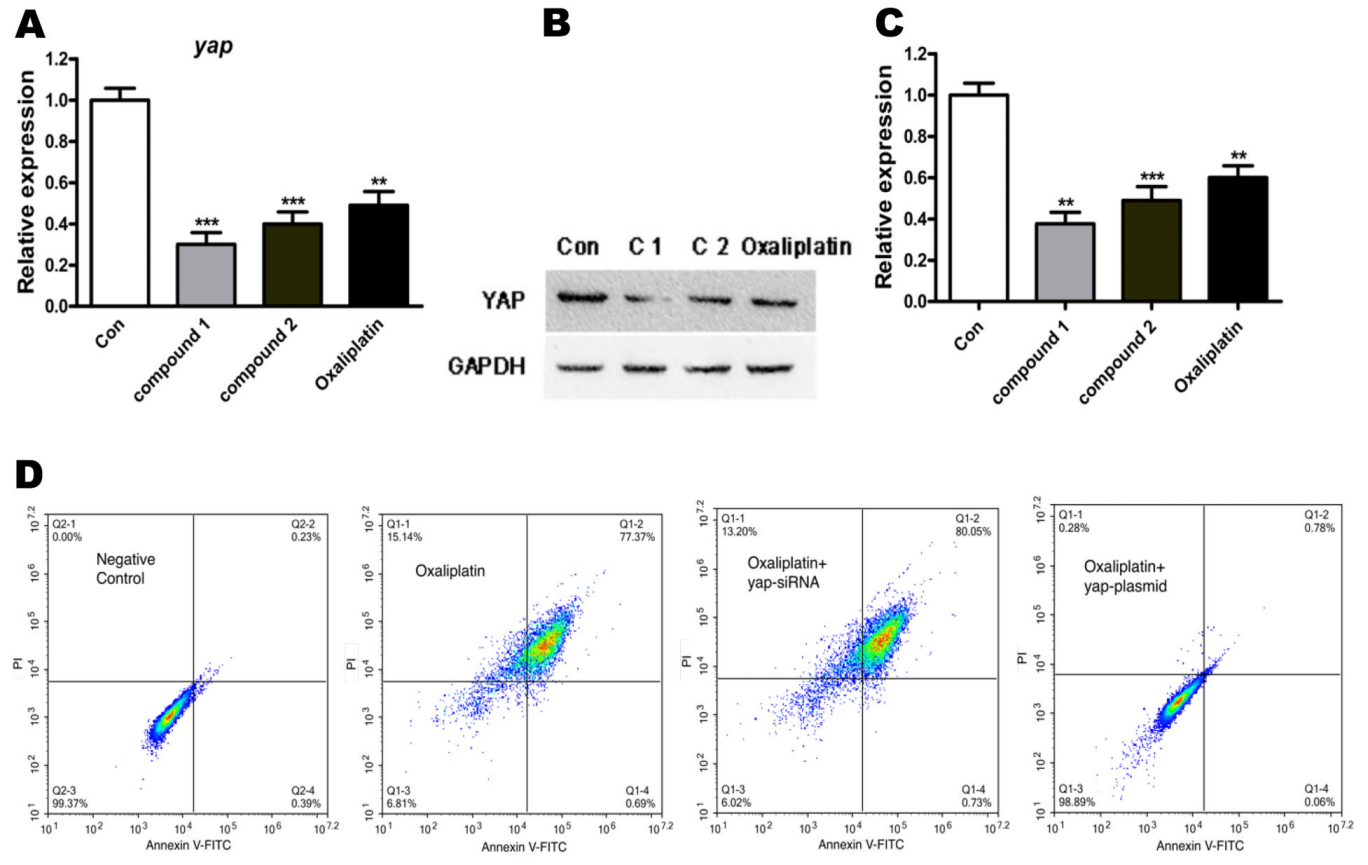
Compounds suppressed YAP expression and induced cell apoptosis. **A**, The *yap* gene mRNA expression was detected with qRT-PCR. **B**, YAP protein expression was analyzed by western blot. **C**, Statistical results of YAP protein expression. **D**, After transfection with *yap*-siRNA or *yap*-plasmid, cell apoptosis was measured by annexin V-FITC/PI staining assay. Data are reported as means±SD. **P<0.01 and ***P<0.001 compared to control (Con) (ANOVA).

As reported, YAP is an important effector in the hippo pathway, which also plays a role in in regulating cell apoptosis. Thus, we speculated that the induced OPM-2 cell apoptosis may have a relationship with the expression level of YAP protein and the activation of the hippo pathway. To test this hypothesis, we firstly detected the *yap* gene expression after treatment with the compounds. As shown in Figure 7A, the *yap* gene expression exhibited a decline, especially when treated with compound 1. The western blot detection shown in Figure 7B confirmed this phenomenon. Next, we explored whether YAP plays any role in regulating cell apoptosis. The *yap* gene was silenced or enhanced with siRNA and plasmid transfection, and the apoptotic cells induced by treatment with compounds were quantified with annexin V-FITC/PI staining assay. The cells in the *yap*-silenced group showed a high basal apoptosis rate (1.8±0.208%). The *yap* overexpression, however, significantly reduced the apoptosis rate to 5.2±1.241% (Figure 7D).

## Conclusion

We have developed novel 1D Zn and Co-based coordination complexes with interesting structural properties that showed significant positive effects over existing anti-tumor systems. The self-assembly of coordination complexes was carried out under mild conditions, making the complexes highly stable. Compound 1 revealed a 1D chain-like network, which was further extended into a 3D surpramolecular network via the H-bond interaction between the H atom on the imidazolyl N atom and the O atom of the bdc^2-^ linker. Compound 2 showed a 1D chain-like network, which was further extended into 2D surpramolecular network via the H-bond interaction between the H atom of the water molecule and the O atom of the sbc^2-^ linker. In the biological study, compounds 1 and 2 inhibited the proliferation of OPM-2 cells in a dose-dependent manner, and the activity of compound 1 was much higher than compound 2. We further revealed that the new synthesized compounds suppressed YAP expression, then led to an increase of apoptotic cell death, and finally exerted the anti-cancer activity on OPM-2 cells. All data from the present study suggested that compared with compound 2, the novel synthesized compound 1 was a more promising candidate for developing anti-cancer drugs targeting human spinal tumor cells.
